# The peritoneum in perspective: extracellular vesicles and the future of peritoneal dialysis

**DOI:** 10.1080/0886022X.2026.2622256

**Published:** 2026-02-04

**Authors:** Natalia Stepanova

**Affiliations:** a Department of Nephrology and Dialysis, The State Institution “O.O. Shalimov National Scientific Center of Surgery and Transplantology of the National Academy of Medical Science of Ukraine”, Kyiv, Ukraine; b Dialysis Medical Center LLC “Nephrocenter”, Kyiv, Ukraine

**Keywords:** Peritoneal dialysis, extracellular vesicles, peritoneum, biomarkers, precision medicine, translational research

## Abstract

Peritoneal dialysis (PD) transforms the peritoneum into a dynamic therapeutic interface, with each exchange offering direct access to molecular and cellular signals from the peritoneal cavity. Among these, extracellular vesicles (EVs) have emerged as stable, information-rich messengers reflecting peritoneal health, inflammation, and fibrosis. The review explores the peritoneum as a living therapeutic interface, summarizing current evidence on EV biology, their molecular cargo, and potential roles in monitoring inflammation, fibrosis, and membrane function. It also discusses existing knowledge gaps, technological advances, and opportunities for translating EV research into clinical practice.

## Introduction

Peritoneal dialysis (PD) is a well-established home-based modality of kidney replacement therapy, providing patients with greater autonomy and flexibility compared to in-center hemodialysis [1]. In PD, the peritoneal membrane serves as a variably permeable barrier, enabling solutes and fluid exchange between the blood and dialysate instilled into the abdominal cavity [2]. Traditionally seen as simply removing toxins and excess fluid, PD effluent actually reflects the activity and health of the peritoneum.

Anatomically and physiologically, the peritoneum is more than a passive exchange surface. It is a biologically active and immunologically responsive tissue, richly vascularized and innervated, with the capacity to participate in immune signaling, respond to injury, and support localized therapeutic interventions [3,4]. Each PD exchange generates liters of effluent enriched with cells, proteins, nucleic acids, and metabolites. Far from being waste, this fluid records the activity of the peritoneum and its ongoing dialogue with the systemic circulation.

Previous research focused on soluble mediators in PD effluent, such as cytokines, chemokines, growth factors, and immune cells [5–8]. These studies advanced the understanding of inflammation, fibrosis, and peritoneal transport. Yet soluble markers are often unstable and capture only a snapshot of local activity [7,9,10]. What remains missing is a signal that is both stable and integrative, one that reflects the broader biology of the membrane.

Extracellular vesicles (EVs) may provide that signal. Released by nearly all cells into the peritoneal cavity, EVs act as both indicators of peritoneal health and active participants in its regulation [11,12]. From this perspective, EVs represent the next step in translating peritoneal biology into clinical practice. They offer new opportunities to monitor the membrane over time, predict outcomes, and potentially serve as therapeutic agents [11,13]. Nevertheless, most EV research in kidney disease examined systemic circulation, renal parenchymal injury, or urinary EVs. The unique setting of PD, with direct access to intraperitoneal EVs, has received far less attention.

This review is the first to provide a focused synthesis of EVs isolated specifically from PD effluent. It explores how EVs emerge from the biology of the peritoneal membrane, reviews current evidence from PD studies, and highlights opportunities they may present for future care. Integrating current mechanistic and clinical insights, the review defines a distinct and underexplored niche within EV research. With evidence still limited, it emphasizes the need for systematic studies to clarify the biology, clinical importance, and translational potential of PD effluent EVs.

## The peritoneum: a living interface shaping effluent biology

**Figure 1.**
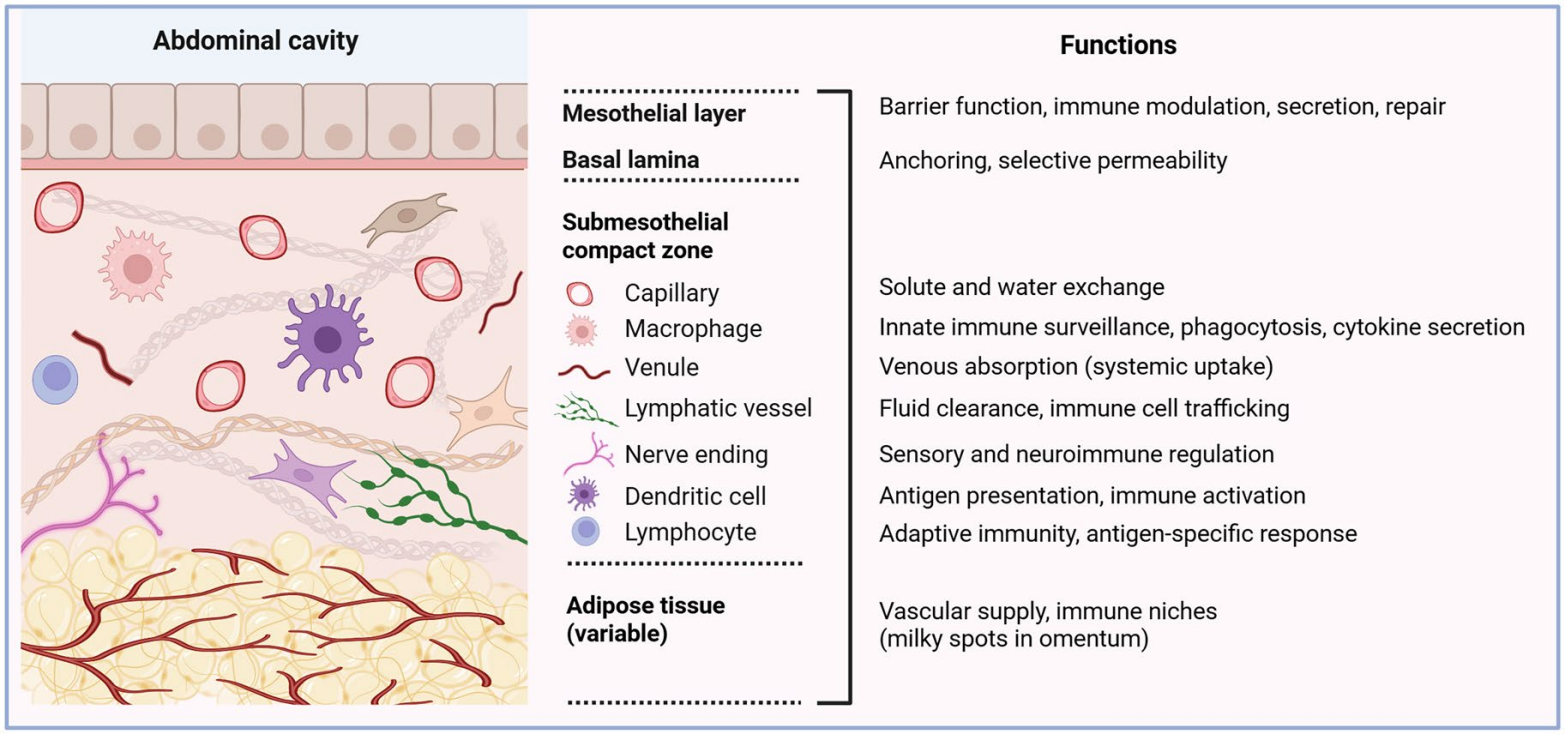
Anatomical structure and functional roles of the peritoneal membrane (created in BioRender.com). Schematic overview of the peritoneal membrane. The mesothelial layer and its basal lamina form the primary barrier, supporting secretion, immune modulation, and selective permeability. The underlying submesothelial compact zone contains vessels, nerves, and immune cells that mediate solute exchange, venous absorption, lymphatic clearance, immune surveillance, and neuroimmune regulation. Deeper adipose tissue, particularly in the omentum, provides vascular supply and immune niches such as milky spots.

Long regarded as merely a simple serous membrane lining the abdominal cavity and its organs, the peritoneum is now understood to be a complex, dynamic, and immunologically active interface with structural, vascular, neural, and immune functions [3,14,15]. Its layered design enables both barrier and regulatory roles, influencing abdominal and systemic health (Figure 1).

In the context of PD, this living membrane is in direct and repeated contact with the dialysate, meaning that every exchange captures molecular and cellular signals originating from the peritoneal tissues themselves. PD effluent, therefore, serves as a noninvasive window into peritoneal health.

At the outermost level, the mesothelial monolayer consists of squamous-like epithelial cells resting on a thin basement membrane [3,16]. These cells establish tight junctions and desmosomes that maintain barrier integrity, while apical microvilli increase surface area and aid in fluid exchange [14,16]. Beyond this structural role, mesothelial cells are immunologically active. They release cytokines, chemokines, growth factors, and EVs that shape the peritoneal immune environment [14,17,18]. Mesothelial cells can also act as nonprofessional antigen-presenting cells, phagocytose particles, and regulate leukocyte adhesion and trafficking [17,19]. During injury, they may undergo mesothelial-to-mesenchymal transition, contributing to repair, fibrosis, and remodeling [14,16]. Many of these mediators and vesicles enter the dialysate, making PD effluent a direct reflection of mesothelial biology.

Beneath the mesothelium lies the basement membrane, composed of collagen, laminin, and proteoglycans. It provides structural support but also acts as a signaling interface, influencing cell adhesion, differentiation, and permeability [3,18]. Alterations at this interface, including fibrosis or increased permeability, are mirrored in the protein and cellular content of PD effluent.

The submesothelial connective tissue offers mechanical stability and houses vascular, lymphatic, and immune elements. Capillaries here mediate bidirectional water and solute transport, while venules return fluid to the systemic circulation [17,20]. Together, these microvascular structures give the peritoneum its extraordinary capacity for bidirectional transport, a property central to both homeostasis and clinical applications such as PD. Lymphatic vessels, particularly in the diaphragm, clear fluid, proteins, pathogens, and debris. They also transport immune cells to regional lymph nodes, linking local activity to systemic responses [21]. As a result, solutes, nucleic acids, and immune mediators moving across this barrier can be sampled in PD effluent.

The peritoneum is also densely innervated. Nerve endings in the submesothelium provide sensory feedback, including pain and mechanical signals [15]. Sympathetic and parasympathetic fibers regulate vascular tone and immune activity, highlighting how the peritoneum is embedded in wider physiological networks [15,22]. Although understudied, these neural signals may also leave measurable traces in effluent, offering possible biomarkers of pain, inflammation, or autonomic activity.

Immunologically, the peritoneum is a distinct compartment that hosts both resident and circulating immune cells [23]. Macrophages dominate, existing as long-lived resident cells and as monocyte-derived subsets recruited during inflammation. Resident macrophages patrol the cavity, maintaining tolerance under steady-state conditions and mounting rapid responses to infection or injury [23,24]. Their polarization toward pro-inflammatory or reparative states shapes outcomes in peritonitis, fibrosis, and PD. Lymphocytes add adaptive capacity: T cells regulate inflammation and cytotoxic responses, while peritoneal B1 cells produce natural IgM antibodies important for early defense [25]. Dendritic cells present antigens and link local immunity to systemic responses, while neutrophils flood the cavity during infection to provide frontline defense [26,27]. Milky spots of the omentum serve as centers for antigen presentation, lymphocyte activation, and coordination of innate and adaptive immunity [28]. Thus, PD effluent contains not only soluble mediators but also intact immune cells, creating a practical means to study human immunity *in vivo*.

The signaling environment of the peritoneum is equally complex. Mesothelial cells, fibroblasts, endothelial cells, and immune populations secrete a wide range of cytokines and chemokines that shape peritoneal inflammation, fibrosis, and angiogenesis. Key mediators include interleukin-6, tumor necrosis factor-α, and transforming growth factor-β (TGF-β), which drive inflammation, tissue remodeling, and angiogenesis [29]. Monocyte chemoattractant protein-1 and interleukin-8 recruit monocytes and neutrophils, respectively [29,30]. Interferon-γ-inducible protein-10 supports T cell trafficking, and stromal cell-derived factor-1 regulates stem and progenitor cell migration [31]. The balance between pro-inflammatory and pro-resolving pathways ultimately determines whether the peritoneum heals successfully or progresses toward chronic inflammation and fibrosis.

However, soluble mediators mentioned above have important limitations. Many are unstable, rapidly degraded, or reflect only a brief snapshot of local activity [5,10]. This makes it difficult to capture the integrated, dynamic biology of the peritoneal membrane over time using soluble biomarkers alone.

In this context, EVs have emerged as promising candidates to fill this gap. Standing out as stable, information-rich messengers [11,12], EVs warrant closer consideration of their biology, molecular cargo, and potential clinical applications.

## Overview of EV biology and functional roles

EVs are nanoscale, membrane-bound particles released by virtually all cell types under physiological and pathological conditions [32]. They represent an evolutionarily conserved intercellular communication system, enabling cells to exchange proteins, lipids, nucleic acids, and metabolites within stable, lipid-enclosed structures [32,33]. In contrast to freely soluble mediators, which are rapidly degraded in biological fluids, EVs protect their cargo from enzymatic digestion and environmental stress, thereby preserving the molecular signatures of their parent cells over time [32,34].

EVs are heterogeneous and are commonly grouped into three major subtypes based on biogenesis, size, and molecular composition [12,32,35]:

- *Exosomes* (30–150 nm): Generated via the endosomal pathway, where intraluminal vesicles form within multivesicular bodies before fusing with the plasma membrane to release their contents.
- *Microvesicles* (100–1,000 nm): Produced by outward budding and fission of the plasma membrane, typically triggered by cell activation, metabolic stress, or injury.
- *Apoptotic bodies* (>1,000 nm): Released from cells undergoing programmed cell death and carrying nuclear fragments, cytoplasmic material, and even entire organelles.

Despite these distinctions, overlapping size ranges and technical challenges in isolation often lead researchers to use the umbrella term ‘EVs’ in clinical and translational studies [36]. Regardless of origin, EVs are enriched in bioactive molecules, including proteins, lipids, mRNA, microRNA, and mitochondrial DNA, that mirror the physiological and pathological states of their parent cells [12,32,35].

Importantly, EV release is not merely a passive process but is tightly regulated by cellular signals. EVs can be released constitutively or in response to environmental stimuli such as inflammation, oxidative stress, or mechanical and metabolic stress [37]. In PD, repeated exposure to hypertonic glucose, glucose-degrading products (GDPs), oxidative stress, mechanical stretch, and recurrent peritonitis activates inflammatory and profibrotic pathways in peritoneal cells. As a direct consequence, mesothelial, endothelial, immune, and stromal cells release EVs into the peritoneal cavity [30,38,39].

Once in the extracellular space, EVs interact with recipient cells via ligand–receptor binding, endocytosis, macropinocytosis, phagocytosis, or direct membrane fusion, enabling transfer of functional cargo and modulation of cellular behavior [37,40]. These routes allow the delivery of functionally active cargo, thereby reprogramming gene expression, metabolic activity, and inflammatory signaling in recipient cells [41,42]. Rising EV concentrations during a single dialysis dwell correlate with markers of mesothelial stress and inflammation [38,43]. *In vitro*, high-glucose or GDP-rich dialysate stimulates secretion of EVs enriched with inflammatory and profibrotic mediators, directly linking peritoneal injury to EV biogenesis [39,44].

Functionally, EVs exhibit context-dependent roles. They carry nucleic acids, proteins, and lipids that can reprogram gene expression, cellular phenotype, and metabolic activity in recipient cells [41,45]. By removing damaged molecules and modulating immune responses, EVs maintain tissue homeostasis and barrier integrity [40]. Conversely, they also mediate inflammation, fibrosis, angiogenesis, and tissue remodeling, propagating either injury or repair signals depending on their cellular origin and cargo [41,45].

**Figure 2.**
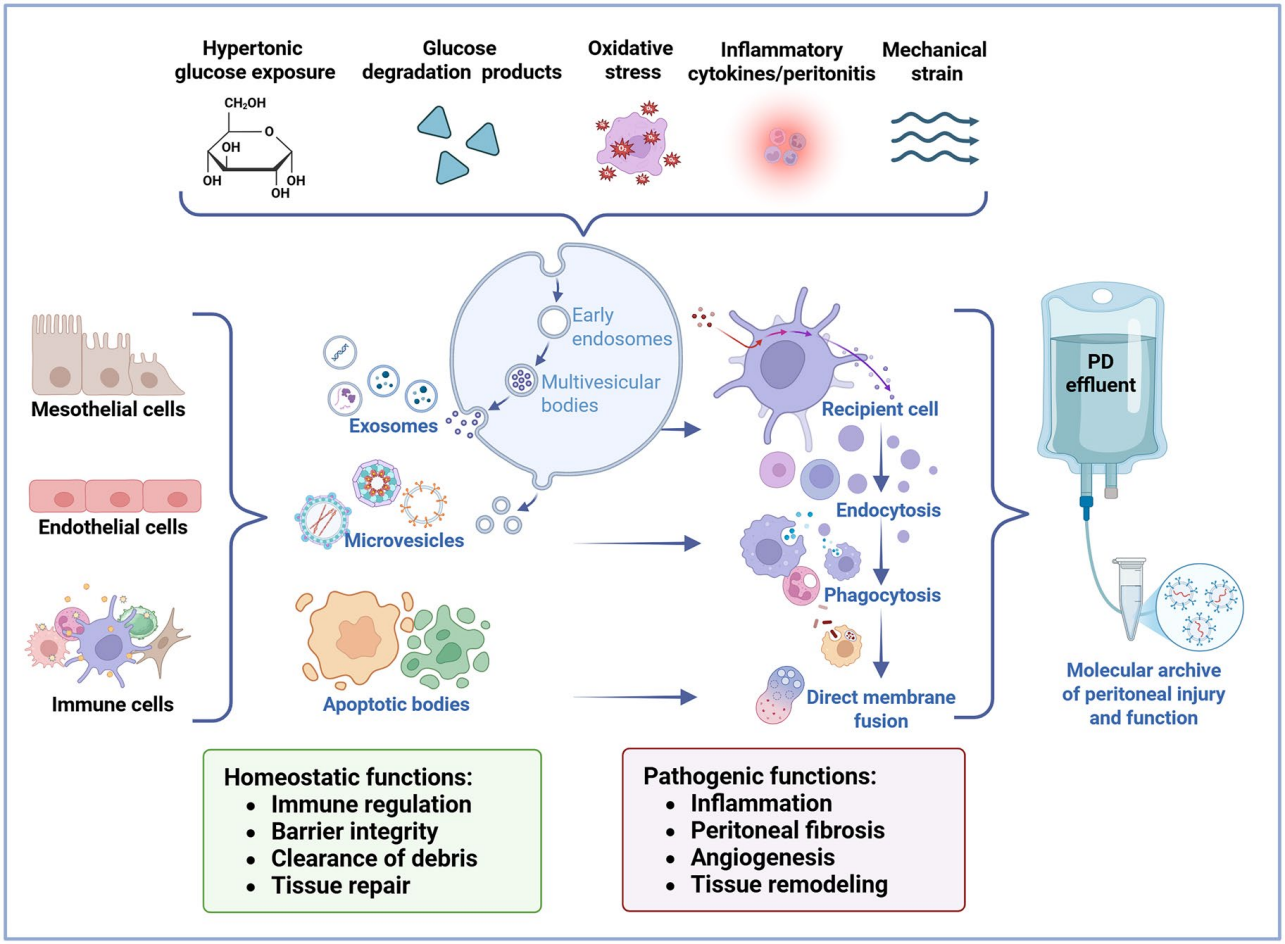
EVs in PD: sources, stimuli, uptake mechanisms, and functional roles (created in BioRender.com). Environmental stressors in PD, including hypertonic glucose, GDPs, oxidative stress, inflammatory cytokines, and/or peritonitis, and mechanical strain, stimulate peritoneal mesothelial cells, endothelial cells, and immune cells to release EVs. Three main EV subtypes are shown: exosomes generated via the endosomal pathway, microvesicles derived from plasma membrane budding, and apoptotic bodies released from dying cells. Once secreted into the peritoneal cavity, EVs deliver bioactive cargo, including proteins, nucleic acids, and lipids, to recipient cells via ligand–receptor interactions, endocytosis, phagocytosis, or direct membrane fusion. Depending on their cellular origin, stimuli, and molecular composition, EVs mediate either homeostatic functions or pathogenic functions. Inflammatory and oxidative stressors tend to promote EVs with pro-inflammatory or pro-fibrotic cargo, whereas more stable conditions can favor EVs involved in barrier maintenance, immune modulation, or tissue repair. Although PD stressors clearly influence EV release, the precise mapping of individual stressors to specific EV-mediated outcomes remains incompletely defined and is illustrated here conceptually. EVs accumulating in PD effluent form a molecular archive reflecting real-time peritoneal injury, inflammation, and functional status, providing a basis for biomarker discovery and potential therapeutic targeting.

Thus, rather than being inert by-products, EVs act as dynamic messengers orchestrating either tissue repair or injury responses, depending on the balance between protective and pathogenic signals in their cargo. As such, they are increasingly recognized as promising biomarkers and potential therapeutic targets for modulating inflammation, fibrosis, and angiogenesis in PD (Figure 2).

## Evidence, knowledge gaps, and methodological limitations in PD effluent EV research

Research into PD effluent EVs is rapidly expanding but remains at an early stage. Several studies demonstrated that EVs can be reproducibly isolated from routine PD effluent, carrying proteins, nucleic acids, and metabolites that reflect the underlying biology of the peritoneum [11,13,38]. Proteomic studies revealed thousands of proteins within EVs in PD effluent, including markers of inflammation, fibrosis, and vascular stress, confirming that these vesicles provide a molecular archive of peritoneal health rather than simple cellular debris [11,38].

A number of molecular signatures now consistently associate EVs with specific peritoneal processes rather than generic cellular turnover. Exosomal miR-432-5p levels correlated with ultrafiltration capacity and small-solute transport rates, suggesting potential as a biomarker of peritoneal function [46]. EVs bearing aquaporin-1 (AQP1), a water channel expressed in endothelial and mesothelial cells critical for transcellular ultrafiltration, were identified in PD effluent and correlated with free-water transport and ultrafiltration efficiency [47]. Conversely, EVs enriched with integrin-linked kinase (ILK) were released from stressed mesothelial cells and activated fibroblast p38 mitogen-activated protein kinase (MAPK) signaling, linking EV cargo to peritoneal fibrosis severity [44]. The percentage of ILK-positive EVs in PD effluent correlated with the degree of peritoneal damage and dysfunction. EVs containing glycoprotein 96 (GP96) were associated with high transport status and systemic inflammation, indicating vascular or endothelial stress responses during PD [39]. GP96 is known to activate Toll-like receptor 2/4 and downstream nuclear factor kappa-light-chain-enhancer of activated B cells (NF-κB) signaling in immune and endothelial cells, providing a plausible link between EV cargo and NF-κB-dependent cytokine release [39,48].

Functional studies align with these molecular associations and provide mechanistic support. *In vitro* and *in vivo* studies showed that PD effluent EVs inhibited epithelial–mesenchymal transition (EMT) induced by TGF-β or platelet-derived growth factor-B (PDGF-B), limited collagen deposition, and preserved mesothelial integrity while reducing submesothelial thickening [44,49]. These protective responses corresponded to reduced phosphorylation of Smad2/3 and extracellular signal-regulated kinase 1/2 (ERK1/2) activation, indicating direct EV-mediated interference with canonical profibrotic signaling pathways [49].

Evidence from mesenchymal stem cell (MSC)-derived EVs in PD injury models further strengthened this interpretation. Bone marrow MSC exosomes delivering miR-27a-3p reduced peritoneal fibrosis by modulating tumor protein 53 (TP53), lowering collagen and α-smooth muscle actin (α-SMA) expression, and constraining Smad activity [50]. Human umbilical cord MSC EVs transferred long non-coding RNAs such as growth arrest-specific transcript 5 (GAS5), inhibited the microRNA-21/phosphatase and tensin homolog (miR-21/PTEN) axis, and suppressed Wnt/β-catenin signaling in high-glucose-exposed mesothelial cells, thereby attenuating EMT and matrix accumulation [44,51]. Collectively, the currently available studies describing PD effluent EVs are summarized in Table 1.

**Table 1.** Current evidence on PD effluent EVs.

Study	Patients/samples	EV Isolation and characterization/biomarker type	Main findings	Clinical/functional relevance	Key limitations
Akbari et al. [43]	8 adult PD patients, new to PD (first PET)	MPs 0.1–1.0 µm isolated from PD effluent; characterized by NTA, flow cytometry (mesothelin marker), Western blot, EM	MPs accumulate progressively during a 4-hour PD dwell; size ∼30–900 nm; mesothelin on surface suggests mesothelial origin	Indicates that mesothelial injury can be tracked by MPs; potential early biomarker of membrane stress	Proof-of-concept; small sample size; single center; no long-term outcome data
Pearson et al. [38]	13 PD effluent samples from 8 adult PD patients	Differential centrifugation + size-exclusion chromatograph; proteomics	Multiple EV subtypes identified; >2,000 proteins profiled	Demonstrates the feasibility of PD effluent EV isolation and biomarker discovery	Cross-sectional; no outcome correlation
Carreras-Planella et al. [11,13]	9 adult PD patients (new PD <10 months: *n* = 4; long PD >18 months: *n* = 5)	UF + size-exclusion chromatograph; flow cytometry, NTA, cryo-EM, LC–MS/MS proteomics	2017: 274 proteins identified, 63 ‘core’ proteins in all samples; 2019: temporal proteomic changes linked to PD duration, ECM and inflammatory pathways	Confirmed reproducible PD effluent EV isolation; EV proteome reflects PD duration/membrane changes	Small sample size; cross-sectional; limited follow-up; no functional outcome validation
Corciulo et al. [47]	30 adult PD patients	Differential centrifugation to enrich exosomes; immunoblotting, ImmunoTEM, ELISA; markers included AQP1, mesothelin, Alix; confirmed absence of endothelial marker CD31	AQP1 is present in mesothelial exosomes in PD effluent and mesothelial cells; AQP1 abundance in PD effluent correlates with UF, free water transport, Na-sieving, suggests intact peritoneal barrier function	Suggests AQP1 in exosomes may serve as noninvasive biomarker of dialysis efficiency, peritoneal barrier integrity, and prediction of UF failure	Cross-sectional design; correlation does not prove causation; no long-term outcome data; single center
Bruschi et al. [52]	12 pediatric PD patients (FSGS *n* = 6 vs. non-FSGS *n* = 6)	Mesothelial exosome isolation; mass-spec proteomics	Distinct exosome proteomes in FSGS vs. non-FSGS	Suggests disease-specific EV signatures in PD effluent	Small sample size; pediatric cohort
Tong et al. [46]	40 PD effluent samples; adult PD patients	Exosomal miRNA profiling (miR-432-5p)	miR-432-5p levels correlate with UF volume and PSTR	First evidence linking PD effluent exosomal miRNA to peritoneal transport metrics	Single biomarker; cross-sectional; needs validation
Fang et al. [39]	60 adult PD patients, stratified by transport status	Ultracentrifugation; proteomics; GP96 analysis	GP96-rich PD effluent EVs associate with high transport status and inflammation	Points to EV proteins as markers and potential mediators	Cross-sectional; methodological variability
Wu et al. [53]	6 adult PD patients (UF failure *n* = 3 vs. UF success *n* = 3)	Small RNA-seq; qPCR validation	PD effluent exosomal miRNAs linked to UF failure	Suggests local peritoneal EV signals reflect membrane dysfunction	Very small sample size; cross-sectional; limited validation
Huang et al. [44]	PD effluent/PLF: 18 samples for proteomics; 64 for ILK+ EV flow cytometry; cells: omentum (*n* = 12) for mesothelial and fibroblast cultures; *in vivo*: mouse PD fibrosis model	Ultracentrifugation; NTA, TEM, WB; 4D proteomics; flow cytometry; single-cell RNA-seq; EV inhibition assays	Mesothelial EVs carry ILK cargo driving fibroblast activation via p38 MAPK; ILK+ EVs are enriched in long-term PD; EV inhibition reduces fibrosis *in vitro* and *in vivo*	Links EV cargo (ILK) to peritoneal fibrosis pathogenesis; identifies ILK+ EVs as biomarkers and therapeutic targets	Modest sample size; acute fibrosis model; translation not yet defined
Karsten et al. [54]	13 adult PD patients	EV characterization; early biomarker exploration	Confirms feasibility in real-world PD workflows	Reinforces translational potential	Preliminary, abstract-level detail
Szebeni et al. [49]	10 pediatric PD patients; primary mesothelial and fibroblast cultures; *in vivo* mouse PD fibrosis model (*n* = 6)	PD effluent EVs isolated; characterized by size, CD63, annexin, CD9, etc., flow cytometry, TEM; uptake assays *in vitro*; *in vivo* PD effluent EV administration in mice	PD effluent EVs inhibit TGF-β and PDGF-B induced epithelial–mesenchymal transition and collagen production in mesothelial cells and fibroblasts; preserve mesothelial layer integrity; reduce submesothelial thickening in mice	First evidence that PD effluent EVs may protect against fibrosis	Pediatric cohort; *in vivo* model is relatively acute; established; mechanistic pathways require further delineation

AQP1: aquaporin 1; CD9/CD63: cluster of differentiation 9/63; ECM: extracellular matrix; EV: extracellular vesicle; FSGS: focal segmental glomerulosclerosis; GP96: glycoprotein 96; ILK: integrin-linked kinase; LC–MS/MS: liquid chromatography tandem mass spectrometry; miRNA: microRNA; MPs: microparticles; NTA: nanoparticle tracking analysis; PD: peritoneal dialysis; PET: peritoneal equilibrium test; PDGF-B: platelet-derived growth factor subunit B; PLF: peritoneal lavage fluid; PSTR: peritoneal small solute transport rate; qPCR: quantitative polymerase chain reaction; RNA-seq: RNA sequencing; SEC: size-exclusion chromatography; TEM: transmission electron microscopy; TGF-β: transforming growth factor beta; UF: ultrafiltration; WB: western blot.

Overall, current evidence suggests that EVs are not passive reflections of peritoneal damage. Their cargo can amplify injury through profibrotic and inflammatory pathways, or conversely promote repair by modulating EMT and extracellular-matrix turnover. These opposing effects appear to be mediated through coordinated regulation of TGF-β/Smad, Wnt/β-catenin, NF-κB, and MAPK signaling cascades. The net outcome likely depends on cellular origin, molecular composition, and the surrounding microenvironment.

However, the current literature remains fragmented. Most studies summarized in Table 1 are small, single-center, and cross-sectional, with substantial variability in EV isolation techniques, analytical platforms, and outcome definitions [38,52]. Only a few incorporated longitudinal sampling, standardized pre-analytical protocols, or clinically meaningful endpoints such as peritonitis, ultrafiltration failure, or technique survival [11]. Heterogeneity in EV isolation and characterization remains a major barrier to reproducibility and cross-study comparison [55]. For example, different centrifugation workflows, size-exclusion approaches, and marker panels can produce very different EV populations and biomarker profiles [56,57]. Normalization strategies also differ widely, with studies variably reporting EV abundance per volume, per protein content, or per particle count [55,56,58].

Furthermore, the cellular origin of many EV subtypes in PD effluent remains poorly defined, limiting efforts to reliably link peritoneal anatomy to molecular signatures [44,47]. Advanced techniques such as single-cell transcriptomics combined with vesicle surface profiling could help resolve cellular origins and functional heterogeneity in future work [59]. Measurement platforms add another layer of inconsistency. Nanoparticle tracking analysis, high-sensitivity flow cytometry, and proteomic assays frequently produce non-overlapping particle counts or protein signatures [57,60]. These inconsistencies stand in clear contrast to the more standardized approaches used in systemic EV research. For example, urinary and plasma EV studies routinely apply reference markers such as CD9, CD63, CD81, ALG-2-interacting protein X, and tumor susceptibility gene 101. They also use transparent pre-analytical protocols and normalize data based on particle counts, protein content, or sample volume [56,57]. These practices reduce variability and improve reproducibility. In contrast, PD effluent EV studies often lack consistent normalization, comprehensive marker validation, or adherence to ISEV/MISEV guidelines [38,52]. Adopting practices from urinary and plasma EV research could substantially improve methodological rigor and enable more meaningful cross-study comparison in PD.

Unresolved physiological questions also limit progress. Whether PD effluent EVs interact with peritoneal lymphatic vessels for clearance or transport to lymph nodes, or whether peritoneal nerves contribute to EV signaling or respond to EV cargo, remains unknown. Although studies in non-PD settings suggest that small EVs can enter lymphatic circulation and reach regional lymph nodes [61], no analogous evidence exists for PD to date. Another unexplored area concerns bacterial EVs, which were described in other infectious contexts [62,63] but have not yet been characterized in PD effluent, even though peritonitis is a leading PD complication. Given their potential to carry pathogen-associated molecular patterns and trigger immune activation, bacterial EVs may represent both diagnostic markers and therapeutic targets in infection-associated peritoneal injury [64,65].

Dialysate-related influences on EV biology remain similarly understudied. Although direct comparative studies are lacking, indirect evidence suggests that dialysate composition likely affects EV release and cargo. In pediatric PD, mesothelial exosome proteins such as protein tyrosine phosphatase type IVA 1 correlated with dialysis vintage and peritoneal transport status, implying that glucose exposure and transport characteristics shaped EV profiles [52]. However, controlled studies comparing EV signatures across different glucose concentrations or biocompatible versus conventional PD solutions are still lacking. Beyond local stimuli, systemic-peritoneal EV exchange also remains largely speculative. Experimental work in mice showed that EVs bearing neutrophil, monocyte, erythrocyte, and platelet markers could be identified in both plasma and peritoneal lavage, suggesting a degree of bidirectional trafficking across serosal surfaces [66]. Comparable data are not yet available in PD, so the contribution of circulating EVs to PD effluent signatures remains uncertain.

At the biological level, it is still uncertain whether EV alterations indicate early injury, reflect established damage, or represent compensatory repair responses, a distinction critical for biomarker interpretation and therapeutic translation.

Taken together, major uncertainties persist due to heterogeneous methodologies, lack of standardized EV characterization and normalization, unclear cellular origins, and incomplete insight into how EVs contribute to peritoneal injury versus repair. These gaps make it difficult to determine whether observed EV signals represent early injury, established damage, or compensatory repair. To provide a consolidated overview of these challenges, Table 2 summarizes the principal methodological limitations and unresolved questions currently shaping the field.

**Table 2.** Current methodological limitations and unresolved questions in PD effluent EV research.

Domain	Limitation/knowledge gap	Unresolved questions
Study design	Small, single-center, cross-sectional	Do EV changes precede, reflect, or follow membrane injury?
EV isolation	Heterogeneous methods	Which isolation workflows produce comparable EV populations across centers?
Quantification	Lack of normalization	What reference markers should be used (protein, particle count, RNA)?
Measurement methods	Nanoparticle tracking analysis/flow cytometry/multi-omics inconsistencies	Which analytical metrics are reproducible across platforms?
Cellular origin	Often inferred, not confirmed	Which EVs arise from mesothelial, endothelial, immune, or stromal cells?
Biological interpretation	Injury vs. repair unclear	Are EVs pathogenic, protective, or both depending on context?
Systemic/dialysate EV effects	Poorly studied	How do dialysate composition and systemic EV influx alter PD effluent EVs?
Bacterial EVs	Not studied in PD	Do bacterial EVs contribute to peritonitis pathophysiology?
Reproducibility	Qualitative EV cargo patterns reproducible; quantitative metrics inconsistent	Which EV features show true biological stability vs. method-driven variation?

Without addressing these gaps, it is difficult to understand whether EV changes precede peritoneal injury, reflect ongoing damage, or represent compensatory repair mechanisms.

## From biology to bedside: future horizons for EV research

Three key messages now define the translational trajectory of EV research in PD. First, feasibility is established: PD effluent EVs can be reproducibly isolated, characterized, and profiled using current technologies [11,13,38,54]. Second, biological relevance is evident: EV cargo reflects peritoneal inflammation, fibrosis, and membrane transport, indicating that EVs actively contribute to peritoneal pathophysiology rather than functioning as passive bystanders [39,46,52]. Third, early therapeutic signals are emerging: some EV populations appear protective, opening the door to vesicle-based interventions in the future [44,49]. The therapeutic landscape for EV research is summarized in Table 3.

**Table 3.** EVs from experimental and stem-cell sources with antifibrotic or reparative effects in PD.

Source	Experimental model	Mechanism/pathway (as reported)	Outcome/effect	Reference
Adipose-derived stem-cell EVs	Rat model of chlorhexidine-induced PD-associated fibrosis	Decreased TGF-β1, α-SMA, collagen I/III; inhibition of macrophage infiltration and angiogenesis	Prevented peritoneal fibrosis and preserved UF capacity	Gouveia et al. [67]
Bone-marrow MSC EVs	*In vitro*: mesothelial models *In vivo*: mouse model of PD-injury	Inhibition of EMT and apoptosis via PI3K/Akt and Smad signaling	Alleviated PD-associated peritoneal injury	Yu et al. [51]
Bone-marrow MSC EVs (miR-27a-3p)	Mouse model of PD-associated fibrosis	miR-27a-3p/TP53 axis, decreased collagen I, fibronectin, α-SMA	Reduced peritoneal fibrosis and improved membrane function	Zhao et al. [50]
Macrophage-derived exosomes ± astragaloside IV	Mouse model of PD-associated fibrosis	Suppressed TGF-β1/Smad2/3 and inflammatory cytokines	Reversed established fibrosis and reduced inflammation	Shan et al. [68]
Umbilical-cord MSC EVs	Human mesothelial-cell senescence and oxidative-stress model	Activation of NRF2/HO-1 antioxidant pathway	Reduced oxidative stress and cellular senescence	Li et al. [69]
PD effluent EVs	*In vitro*: mesothelial and fibroblast cultures *In vivo*: mouse model of chlorhexidine-induced PD-associated fibrosis	Inhibited TGF-β/PDGF-B-induced EMT; limited collagen synthesis; protected mesothelial layer	Decreased submesothelial thickening *in vivo*	Szebeni et al. [49]
Human umbilical cord MSC-derived small extracellular vesicles (exosomal lnc-CDHR)	*In vitro*: mesothelial cells exposed to high-glucose or TGF-β; *in vivo*: mouse PD-fibrosis model	Activated AKT/FOXO signaling; reduced AKT phosphorylation and EMT markers (α-SMA, collagen I)	Attenuated peritoneal fibrosis and preserved mesothelial phenotype	Jiao et al. [70]

α-SMA: alpha-smooth muscle actin; AKT: protein kinase B; CDHR: cadherin-related gene (target of long non-coding RNA lnc-CDHR); EMT: epithelial–mesenchymal transition; EVs: extracellular vesicles; FOXO: forkhead box O; HO-1: heme oxygenase-1; lnc-CDHR: long non-coding RNA CDHR; miR: microRNA; MSC: mesenchymal stem cell; NRF2: nuclear factor erythroid 2-related factor 2; PD: peritoneal dialysis; PDGF-B: platelet-derived growth factor B; PI3K: phosphatidylinositol 3-kinase; sEVs: small extracellular vesicles; Smad: small mothers against decapentaplegic; TGF-β: transforming growth factor β; TP53: tumor protein p53; UF: ultrafiltration.

These studies, spanning mesenchymal, adipose, and immune cell-derived vesicles, demonstrate antifibrotic, anti-inflammatory, and antioxidant mechanisms that may be harnessed for peritoneal repair. Collectively, they provide a promising preclinical foundation for translational progress in PD.

However, converting these experimental results into clinical practice will require stepwise validation and realistic expectations about what can be achieved in the near term. To provide this perspective, the distinctions between applications that are clinically attainable in the near future and those that remain exploratory are summarized in Box 1.

Box 1. Near-term achievable applications vs. exploratory avenues in PD effluent EV research.

**Table:** 

*Near-term, realistically achievable (next 3–5 years):* *Biomarker identification and validation* for inflammation, membrane transport, and early fibrosis (e.g., AQP1, GP96, miR-432-5p, and miR-27a-3p) *Standardized EV isolation and measurement workflows* across PD centers *Longitudinal EV profiling integrated with routine PD assessments* (UF capacity, PET, peritonitis) *Advanced multi-omics characterization* (proteomic and RNA signatures) linked to clinical outcomes *Machine-learning models* incorporating EV data for risk stratification
*Exploratory/longer-term (experimental, requires major development):* *Therapeutic EVs* (MSC-derived, macrophage-derived, engineered EVs) *EV-based drug delivery systems* targeted to the peritoneum *Selective inhibition of pathogenic EV subsets* (e.g., ILK-enriched vesicles) *On-site point-of-care EV quantification* during each PD exchange *Real-time EV biosensors on cyclers* for automated monitoring of membrane health

AQP1: aquaporin-1; EV: extracellular vesicle; GP96: glycoprotein 96; ILK: integrin-linked kinase; miR-27a-3p: microRNA-27a-3p; miR-432-5p: microRNA-432-5p; MSC: mesenchymal stem cell; PD: peritoneal dialysis; PET: peritoneal equilibrium test; PSTR: peritoneal small solute transport rate; UF: ultrafiltration.

In moving toward translational implementation, the next phase of research should focus on coordinated, multi-center efforts applying standardized methodologies and evaluating EV signatures across the clinical course of PD. Well-designed longitudinal cohorts should incorporate serial sampling at PD initiation, during stable treatment, and following complications to clarify whether EV profiles predict outcomes or reflect established injury. Interventional trials testing whether modifications in PD prescriptions, antifibrotic treatments, or infection-prevention strategies alter EV signatures would provide critical mechanistic insight.

At the same time, technological innovation offers new opportunities. Multi-omics platforms, single-cell profiling, and spatial transcriptomics can map the origins and functional diversity of EV populations [71]. Biosensors, microfluidics, and point-of-care diagnostics could enable real-time measurement of EV biomarkers during routine PD exchanges. Integration with artificial intelligence and machine learning can turn these data streams into predictive tools for risk stratification and individualized therapy [71]. Collaborative efforts, including shared biobanks, harmonized analytic pipelines, and centralized data platforms, will be essential to validate findings across centers, facilitate meta-analyses, and accelerate clinical translation [72,73].

Finally, therapeutic translation should be a priority. Harnessing protective EV populations, blocking pathogenic ones, or engineering vesicles as drug carriers could transform PD from a reactive therapy into a precision, preventive strategy. Preclinical work in oncology, cardiovascular disease, and regenerative medicine already demonstrates that engineered EVs can deliver nucleic acids, proteins, and small-molecule drugs to target tissues [34,74,75]. Safety considerations, including off-target effects, immune activation, and manufacturing standards, will be critical before clinical application in PD can be envisioned. Large-scale EV production and storage are technically feasible, suggesting that similar strategies may be adaptable to peritoneal delivery once efficacy and safety are confirmed [73,76].

## Conclusions

PD effluent offers a unique, noninvasive window into the biology of the peritoneal membrane, capturing signals of injury, inflammation, and repair. Among its components, EVs have emerged as stable, information-rich messengers that encapsulate molecular signatures of peritoneal health and disease. Evidence summarized in this review links EV profiles to peritoneal inflammation, fibrosis, ultrafiltration efficiency, and membrane remodeling, while experimental studies indicate both pathogenic and protective roles depending on cellular origin and molecular cargo.

The current body of work demonstrates the feasibility of isolating and characterizing EVs from PD effluent and highlights their biological relevance as markers of local pathophysiological processes. At the same time, it reveals substantial heterogeneity in methodologies and study designs, with most investigations limited to small, cross-sectional analyses. Together, these findings establish EVs as key indicators of peritoneal status and lay the groundwork for integrating EV biology into the broader understanding of peritoneal membrane function.

Looking ahead, the field now requires a focused and coordinated translational effort. Three priorities emerge as most critical: (i) development and adoption of standardized EV isolation, characterization, and normalization protocols across centers; (ii) longitudinal studies with clinically meaningful endpoints to determine whether EV changes precede, reflect, or predict membrane injury and technique failure; and (iii) exploration of currently overlooked contributors, including bacterial EVs and systemic-peritoneal vesicle exchange, to refine mechanistic understanding and diagnostic potential. Progress in these areas will be essential to define whether PD effluent EVs can evolve from research tools to clinically meaningful biomarkers and therapeutic candidates.

## Data Availability

No new data were generated for this review. All data supporting the findings of this work are available in the published literature and can be accessed through the references cited in the manuscript.
